# Racism, Empire and Sociology

**DOI:** 10.1177/0038038516641856

**Published:** 2016-04-29

**Authors:** Andrew Smith

**Affiliations:** University of Glasgow, UK

‘[T]he entity called Europe was constructed from the outside in as much as from the inside out.’ So wrote Mary Louise Pratt, at the start of *Imperial Eyes* (1992: 6), her insightful account of colonial travelogue. Pratt’s point provides a pithy summary of one of the central claims of what was, at the time, the still emerging field of postcolonial studies (in which context, of course, *Imperial Eyes* was a path-breaking text). This is the claim that the practices and the relations of empire need to be acknowledged as constitutive of European self-understanding. Far from empire being something which lay beyond, outside or apart from Europe it was, to a considerable extent, in and through imperial practices and relations that Europe came to recognize and define itself. That recognition has led to a wide-ranging critical re-evaluation within a range of disciplines. English literature, for example, has seen important accounts of its disciplinary emergence from within the pedagogical contexts of empire (e.g. Azim, 1993; Viswanathan, 1987), and of the ways in which conventional literary studies have reflected and reproduced the forms of knowledge and the self-identities on which empire depended. Yet, as Gurminder Bhambra argues, in the first volume of the new Bloomsbury monograph series, ‘Theory for a Global Age’, sociology has been remarkably reluctant to engage in any correspondingly critical reflection on its own relationship to the history and conceptual legacies of empire. Bhambra’s *Connected Sociologies*, along with a subsequent volume from the same series – Peo Hansen and Stefan Jonsson’s *Eurafrica –* and a new title from Verlag’s series on ‘Racism Analysis’, edited by Wulf D Hund and Alana Lentin – *Racism and Sociology* – provide a welcome and necessary reiteration of the need to rethink Europe through the histories of empire, and a no-less necessary reiteration of the need for sociology to re-examine its own epistemological claims in the shadow of that same history (see also Go, 2013; Steinmetz, 2013).

Bhambra, restating and developing the argument she made initially in *Rethinking Modernity* (2007), emphasizes the extent to which sociology, according to its own canonical story and by virtue of an intellectual division of labour which assigned to anthropology the study of ‘other’ cultures and places, was (and is) constituted as the science whose proper object is ‘modern society’. In this account modernity is construed as a thing-in-itself, something with unique and uniquely potent historical characteristics. In this respect, then, ‘sociology’s self-understanding [is] brought about in the European production of modernity as distinct from its colonial entanglements’ (p. 2), such that the violently authoritarian forms of power on which colonial rule routinely depended, and the racialized structures of inequality which it established, are seen to fall outwith the purview of proper sociological inquiry. The tendency then, Bhambra argues, of even those sociological traditions which approach modernity as an object of criticism rather than celebration, has been to separate ‘historical injustices from any consideration of justice in “modern” societies’ (p. 145). Put otherwise: modernity is construed as a puzzle to be solved ‘from the inside’, a process whose explanation can be sought within allegedly self-contained European social developments (the political, scientific and industrial revolutions, etc.), and whose global effects are only ever subsequent to those internal developments. Insofar as it represents itself as the expression of modernity’s self-understanding, therefore, sociology becomes the epistemological guarantor of the idea of Europe as an autonomous historical totality. By the same token it serves to make possible the discounting of the histories of colonialism as extrinsic or incidental factors that need not trouble the self-portrait of ‘the West’ as the cradle of Enlightenment, democracy and historical progress.

Bhambra traces the stubborn resilience of this story through a range of sociological attempts to conceive of ‘the global’, starting with familiar classical precedents and moving through pointed critiques of modernization theory, underdevelopment theory, Eisenstadt’s ‘multiple modernities’ model and the ‘world historical’ accounts of Braudel and Wallerstein. She then follows the repercussions of the same presumptions in more recent moves to inaugurate cosmopolitan, global or ‘indigenous’ sociologies: even these latter, she argues, tend towards an ‘additive’ approach. In other words, the move to open up the sociological canon so as to include new standpoints and perspectives, insofar as such moves are represented as a response to *newly* emergent historical conditions, can become a kind of alibi by which ‘earlier global interactions constituted via processes of colonialism, imperialism and slavery are effaced from consideration’ (p. 112).

It seems crucial to me that, as a discipline, we take this demand for critical self-reflection on our ‘modes of knowing’ seriously. But perhaps some important resources are dismissed a little too quickly? Bhambra’s final call is for a reformulated historical sociology which rejects the construction of differing civilizations or cultures as self-contained, and begins instead from ‘the histories of interconnection that have enabled the world to emerge as a global space’ (p. 155). To me, at least, this move does not seem quite as far away from the original intention of dependency or world-systems theory as is suggested, and the claim that the former failed to connect current inequalities to colonialism (e.g. p. 143) is surely overstated. Certainly there are contributions to those traditions, not least among the so-called ‘articulation’ theorists, which are absolutely concerned to explore the detailed historical connections by which global forms of economic inequality were established and maintained. For most writers in those traditions, of course, capitalism was a necessary part of the explanation in that regard, and this raises a question that might also bear further discussion. As Bhambra absolutely rightly points out, accounts of European modernity as ‘unique’ frequently plead a kind of ‘ideal type’ methodology, behind which lie hidden implicit value judgements about cultural or intellectual superiority: ‘the ideal type of European modernity … is established on the basis of a selection of historical narratives that simultaneously presents a normative argument about European progress and superiority’ (p. 147). That critique – of the assumption of European ontological superiority – is urgent, but it is important also that the object of that critique is not conflated with an analysis of the particular conditions which enabled Europe to extend and enforce unequal relations across much of the world. Reinstating empire in our conceptions of the global implies a need to explore the factors which allowed imperial relations to take the shape they did. While those factors were not simply intrinsic to Europe, they did include the emergence of economic and institutional forms and technologies over which (some) Europeans were able to exert particular control, and from which they derived particular forms of power and advantage. Work in the Marxist theorization of imperialism (much of it established and developed outside of Europe, of course) seems to me to offer an important contribution to our understanding of those processes and connections. It is true that not all of that work is free of the tendency to read unequal historical relations as evidence of some kind of inherent European superiority. At the same time, much of it was profoundly committed to understanding the nature of the processes by which certain kinds of strategic superiority were established and consolidated without falling into the tendency (which postcolonial studies have not always avoided), of seeking explanations only within the world of epistemology and cultural self-understanding. Aijaz Ahmad’s response to Edward Said’s thesis remains cautionary here. What gave European prejudices ‘their special force in history’, Ahmad (1992: 184) insists, was not ‘some gathering of unique forces in the domains of discourse – but, quite specifically, the power of colonial capitalism, which then gave rise to other sorts of powers’.

Bhambra ends by arguing that a ‘new understanding of the global cannot simply be asserted, but has to be argued for in terms of how it addresses the deficiencies and limitations of previous understandings and how it enables more productive insights in the future’ (p. 156). She offers, in the last dozen pages of the study, a short and compelling example in this respect, demonstrating the way in which categories of citizenship and belonging might be reconceptualized through an awareness of the effective political communities that empire established. For the most part, however, her study does not engage in all that much exemplification, but offers instead a necessary clearing of space which works by making the lineaments and limitations of dominant understandings of ‘the global’ explicit. One potential empirical direction in which a ‘connected’ history of Europe might emerge, then, is provided by Hansen and Jonsson’s exceptional study *Eurafrica*, which works from exactly the perspective proposed by Bhambra: ‘in order to think theoretically about globality today’, they argue, ‘it is fundamental to know how the global was conceived in the past’ (p. 277). More specifically, Hansen and Jonsson’s account provides a challenge to the conventional historiography of twentieth-century European integration which, they suggest, tends to explain that process either as the outcome of a post-war reconciliation occurring wholly within the halls of European political diplomacy (‘a foundational tale of pure origins, of an Immaculate Conception’ (p. 3)) or, insofar as wider factors are accounted at all, as a development which responded only to the geostrategic context of the cold war.

What Hansen and Jonsson recover, then, is the occluded history of the extent to which ‘efforts to unify Europe systematically coincide[d] with efforts to stabilize, reform and reinvent the colonial system in Africa’ (p. 6). Starting in the period immediately following the First World War they trace the emergence and endurance of what came to be described as ‘Eurafrica’: that is, a projected unity in which European nations sought to overcome their rivalries by establishing a shared, cooperative exploitation of African territory and resources. Such projects had their utopian or visionary expressions, most dramatically in Herman Sörgel’s proposal for a new transcontinental landmass, Alantropa, to be created by the damming of the Mediterranean. For the most part, though, the account provided here is concerned with more than ideas; Eurafrica was not just imagined, but was also a concrete political project, the details of which the authors recover by a detailed and scrupulous examination of the records of diplomatic negotiation and policy making. Hansen and Jonsson note: ‘a refutation of the EU’s image of itself and of its historical relation to Africa here emerges through the explicit and eloquent wealth of the historical archive itself’ (p. 12).

Their account begins in the period immediately post-World War One which, they note, had ‘brought the conflicts of global imperialism back to Europe’ (p. 17) – something which, we might recall, WEB Du Bois (1915) recognized at the time. In this period, Hansen and Jonsson demonstrate, plans to establish new forms of interstate cooperation were continually formulated in, through and with plans to consolidate new forms of collaboration and agreement as regards colonialism in Africa. Such schemes were motivated, in part, by growing European indebtedness and, particularly after World War Two, by a desire to escape a reliance on dollar imports: Paul Reynaud, French Prime Minister and chair of the Council of Europe’s Committee on Economic Questions, was doing little more than stating the established wisdom – shared by Ernest Bevin, among others – when he said: ‘we must … if free Europe is to be made viable, jointly exploit the riches of the African continent, and try to find there those raw materials we are getting from the dollar area and for which we are unable to pay’ (p. 114). At the same time, however, especially in the face of increasingly assertive decolonization struggles and French defeat in what was then Indochina, Eurafrica was pursued as a means of forestalling African demands for independence, while simultaneously re-establishing European imperial authority on a scale that would allow it to compete with American and Soviet power. Thus key figures in the negotiations which led up to the Treaty of Rome, such as Christian Pineau, insisted that the integration of French overseas territories within Europe’s proposed new political architecture offered a country such as Algeria ‘the true condition of independence’ (p. 215). In practice, as the authors demonstrate, the terms of this proposed integration were profoundly unequal, and included a careful and surreptitious series of moves which ensured that non-European Algerians, for example, would be excluded from free movement provisions. Nevertheless, it was in this way, Hansen and Jonsson argue, that Eurafrica can be read, historically, as a ‘vanishing mediator’. In other words, it allowed for ‘regional integration and consolidation of Europe’s control of Africa, but without having to carry the blame for colonial exploitation and explicit white supremacy’ (p. 255). Although, in the long run, Eurafrican schemes did not come to pass in the way in which they had been originally envisaged, they helped change ‘the ways in which the world system and especially relations between Europe and Africa were described’, while allowing much of the existing relationship of ‘trade, traffic and power to remain unchanged’ (p. 255).

The substantiation of that last point – that is, the ways in which Eurafrican arrangements helped consolidate forms of neo-colonial relationship and served to undercut moves towards pan-Africanist cooperation among the newly independent African nations – is largely deferred to a subsequent volume. Nevertheless, Hansen and Jonsson’s study is invaluable in recovering the imperial history of Europe *qua* Europe: both the extent to which a ‘collaborative colonialism’ was actively pursued among the founder members of the European project and, correspondingly, the extent to which European unity becomes conceivable in and through that pursuit. The key theoretical lessons which Hansen and Jonsson draw from their account emphasize especially the blinkered quality of nationally or regionally focused historiography. They do not engage, therefore, all that much with existing theories of imperialism although there are potentially interesting questions raised by their work in this regard. Karl Kautsky, we might recall, had predicted the emergence of what he called ‘ultra-imperialism’ in the years prior to the First World War – that is, the rise of cooperative forms of global exploitation between existing imperial powers – and Hansen and Jonsson’s study raises rich and provocative questions about how we understand empire and its organization, and about the extent to which national bourgeoisies, in the face of an emerging politics of decolonization, pursued forms of mutual accommodation as a means of entrenching their colonial domination elsewhere.

Among the whole-hearted advocates of the Eurafrican project that Hansen and Jonsson cite (many of whom, including ED Morel and Wladimir Woytinsky, were on the European left), is Raymond Aron. Writing in the context of the Algerian revolution, Aron was explicit in arguing that ‘Algeria is the indispensible southern base of Western Europe; it is the access to the oil in the Sahara’ (p. 185). His view emerges, of course, from a long tradition in French social thought, which can be traced back at least as far as Tocqueville, and which saw in North Africa the prospect of an otherwise-absent ‘frontier’ which would protect and ensure the interests of French democracy (see Smith, 2012). At the same time, however, Aron’s view is an example of one further consequence of sociology’s historical entanglement with European imperialism; that is, the extent to which the discipline has been historically complicit with racism. It is this issue which is the focus of the essays gathered and edited by Wulf D Hund and Alana Lentin in the final book considered here.

Hund and Lentin’s collection, *Racism and Sociology*, addresses both the history of sociology’s contribution to European and North American racism and the contemporary repercussions of that complicity. The more historically focused essays include Hund’s brilliant and wide-ranging survey of racism in classical social theory. Hund shows just how pervasive were conceptions of racial hierarchy in early precursors of sociology such as the Abbé Sieyes and the Scottish moral philosophers, and explores their further elaboration in Britain (Spencer), America (Fitzhugh) and Germany (Weber). In so doing he emphasizes two points. First, he notes that it was quite possible for a figure such as Adam Smith to make use of the racialized categories of empire (e.g. ‘savages’ versus ‘civilized’) in his theoretical work, without any explicit reference to ‘race’ as such: ‘[r]acist discrimination could cope without races from the beginning’ (p. 36). In the same way, Hund demonstrates that Max Weber’s scepticism regarding the ontological validity of ‘race’ as a category did not prevent him from making use of naturalized conceptions of Slavic deficiency, or from elaborating a teleology of development which helped buttress the presumption that Europe occupied the right side of a line of ‘civilizational apartheid’ (p. 54). Second, and very valuably, Hund shows how in the case of Weber and a number of the other thinkers that he considers, racial difference – implicitly or explicitly described – was employed as a means of stilling contemporary fears about social cohesion. It is through the summoning-up of racialized difference that the imagined community of the imperial nation is consolidated: racism serves to resolve the contradictions of class by superimposing ‘a promise of community guaranteed by the shared contempt of others’ (p. 57). Hund’s essay thus substantiates a central claim made by Bhambra, and one can see here how the very social problems (and responses to these problems) which come to be construed as the exclusive domain of sociological inquiry bore within themselves the imprimatur of empire and its ideologies. Colonial racism was always two-sided, of course: the savage could always become the noble-savage and vice-versa. Felix Lösing’s later essay is therefore helpful: his more detailed consideration of one specific and significant figure in the sociological tradition, Robert Park, carefully unpacks the cross-hatching of romanticism and racism which allowed Park to defend the idea of an elevated, civilizing imperialism in Africa during his time with the Congo Reform Association and which led, later, to his nostalgic imagining of black rural life in the USA and his subsequent defence of Jim Crow racism.

Despite these histories, Hund notes, sociology continues to discuss, teach and draw upon many of these theorists unproblematically. In so far as their racism may be acknowledged, it is ‘transferred to the nominee account of the zeitgeist’ (p. 25). That evasion is perpetuated in a number of ways contemporarily, and it is the subject – with different inflections – of a number of the other essays here. Alana Lentin, for example, examines mainstream research on migration and ethnicity in Europe, and the extent to which such research approaches racism, if at all, only as something aberrant or pathological, such that the structuring effects of historical racism are placed outside of inquiry. One trajectory, in this respect, is the limiting of ‘race’ to a narrowly biological definition which, it is presumed, has been safely consigned to the past; another is the treatment of racism as inconsequentially ubiquitous, everywhere and nowhere at once. Much like Bhambra’s conclusion to *Connected Sociologies*, Lentin notes that the ‘given’ categories of migration research – citizen/migrant – reflect claims of identity and belonging which emerged from a racialized and racializing definition of what it was to be European (Hansen and Jonsson’s study, of course, provides valuable insight into the concrete political formation of these categories). Correspondingly, Silvia Rodríguez Maeso and Marta Araújo’s essay reflects on the practical and methodological challenges of undertaking research in such a context, and the importance of turning the focus of empirical inquiry away from the descriptive study of ‘attitudes’ or ‘interactions’ (in a way that presumes that the ‘sociological problem’ is the ‘so-called *presence* of “others”’ (p. 216, emphasis in original)), and onto historically established ‘configurations of racism’ and the ways in which these continue to structure policy, practice and the priorities of funding bodies.

Both essays suggest, then, the importance of paying attention to the continued, structuring effects of ‘race’, but also of thinking critically about the conceptual assumptions at play in the term ‘racism’ itself. It is in just this sense that Barnor Hesse’s essay explores the history by which ‘racism’ was established as a ‘problematic’ in sociology. The intellectual trajectory which Hesse traces has its roots in the immediate post-war response to Fascism among European intellectuals. In that context, Hesse argues, racism comes to be equated with extremism (and is thus presented as aberrant, as a departure from the ‘normal’ liberal practice of European and North American democracy). By contrast, Hesse argues, a hard won ‘black analytics’ has consistently proposed a different understanding, in which racism is recognized not as ‘extreme’ but as routine, as structural rather than ideational, and as historically rooted in empire. Unless we understand the ‘alterity’ within racism – that conceptual otherness at play within the term – we too easily allow a kind of ritualized objection to racism to displace attention from the ‘historical and contemporary … colonial-racial order of the West’ (p. 143). That claim seems crucially important to me, but I find Hesse’s later assertion that ‘[t]he Second World War was not particularly concerned with antisemitism’ (p. 155) more problematic. In saying this, Hesse draws on arguments which trace the origins of Nazi eugenics to antecedents in European colonialism, such that the conflict – and the forms of anti-racism attendant upon it – are interpreted as attempts to re-establish a hegemony of the ‘liberal-colonial-racial order of whiteness’ (p. 155) which had been destabilized by the way in which Fascism replayed imperial racism within Europe. My concern with this interpretation, however, is that it risks flattening out the plurality of histories of racialization. Those histories include, of course, the racialization of communities within Europe and, in a certain respect at least, ‘within whiteness’ – Jews, Irish, Gypsies – which have had their own modalities and which are not always, or not only, explicable in terms of the relations of empire.

Sirma Bilge’s essay, finally, addresses the writing-out of racism in intersectionality scholarship. Powerfully and provocatively, Bilge argues that there has been a recuperation of intersectional approaches, in keeping with what she calls ‘capital’s new interest in particularity’ (p. 181), and neoliberalism’s success in recuperating counter-hegemonic forms of knowledge. The whitening of intersectionality, she argues, is typical of the ‘add-and-stir approach’ by which ‘minority issues’ are incorporated as ‘subject matter to extant disciplines’ (p. 183). Bilge’s response is to call for an ‘epistemic disobedience’, and for a ‘decolonial intersectionality’ – in other words, for an approach which is explicitly committed to a rethinking of itself in the light of the challenges of different standpoints, rather than claiming for itself a kind of curatorial authority over those standpoints. Her call chimes with the reflection on WEB Du Bois and Stuart Hall which is provided, in their essay, by Les Back and Maggie Tate. Both Du Bois and Hall, the authors demonstrate, were profoundly attentive to, and contributed to, developments in mainstream sociology in the periods in which they worked, yet those contributions and interlocutions (as, for example, between Stuart Hall’s notion of conjuncture and C Wright Mill’s concern to understand the relation between the personal and the political) were (and are) rarely allowed to trouble mainstream sociology’s sense of itself. Rather, racism is filed away as a particular and self-contained problem: a ‘mere topic lost within the proliferations of sub-specialisms’ (p. 125). Moreover, Back and Tate point out, both writers did indeed practise a form of ‘epistemic disobedience’, drawing on a range of different ways of telling and writing, and intended to address a broader and more inclusive audience. Like Bilge, Back and Tate recognize that the audit culture of the neoliberal university, and the ‘narrowing of professionalization’ (p. 137), has the likely effect of closing down the very space which the transgressive sociology of a Du Bois or a Hall sought to open out.

Taken all together, then, and alongside Bhambra’s intervention, these studies amount to a call for a sustained re-evaluation of sociology as a discipline and of the historical preconceptions which it bears and tends to reproduce in its ‘telling’ of itself. Bilge suggests in her essay that the very construction of sociology as a science is a part of the problem here – that to be accepted as a sociologist means to ‘abide by the white standards of science’ (p. 186) – and that the pursuit of scientific legitimacy is therefore ‘at odds with the pursuit of social justice’ (p. 202). For what it is worth, such a move would seem to me to give up on the very possibility that we need to defend. As Pnina Werbner (1997) has argued, we can adjudicate between the account of the racist and that of the anti-racist precisely because one is more truthful than the other. In that sense the demand that, as a discipline, we become more attentive to the history and politics of the knowledge which we produce, more attentive to the ways in which the practice and teaching of sociology may carry within it the epistemologies of empire, surely implies a renewed commitment to the central principles of scientific practice: that is to say, a renewed commitment to truthfulness and to the substantiation of our claims through the evaluation of evidence (a la Hansen and Jonsson). It means also, of course, a consistent and critical reflexivity about how our own positioning affects what we see and how we see it: an awareness of the ‘net of constraints’, as Stuart Hall (1986: 42) put it, from within which we go about the business of ‘practical thought and calculation about society’. These commitments, I would argue, rather than being the means of reconsolidating disciplinary authority, may be just the means of reckoning with the dangers of such authority. It is in exactly this regard that the studies considered here make a profoundly welcome contribution.
